# Endocrine Disruptors as a New Etiologic Factor of Bone Tissue Diseases (Review)

**DOI:** 10.17691/stm2021.13.2.10

**Published:** 2021-01-01

**Authors:** N.V. Yaglova, V.V. Yaglov

**Affiliations:** Head of the Laboratory of Endocrine System Development, Research Institute of Human Morphology, 3 Tsyurupy St., Moscow, 117418, Russia; Chief Researcher, Laboratory of Endocrine System Development, Research Institute of Human Morphology, 3 Tsyurupy St., Moscow, 117418, Russia

**Keywords:** endocrine disruptor, bone tissue, bone tissue regeneration, osteoblasts, osteoclasts

## Abstract

At present, diseases of bones and joints stand third after cardiovascular and oncological pathologies which demands the necessity of searching for new etiological factors and pathogenetical mechanisms of these illnesses. The accumulating data show the association between the impairment of bone tissue development and regeneration and endocrine disruptor impact.

Endocrine disruptors are chemical substances, mainly of anthropogenic origin, capable of affecting endocrine system functioning and interfering with organ morphogenesis and physiological functions. The development and regeneration of bone tissues have a complex hormonal regulation and therefore bone tissue cells, osteoblasts, and osteoclasts can be considered as potential targets for endocrine disruptors. Endocrine disruptors have been established to be able to impair calcium metabolism which also contributes to the development of musculoskeletal system pathology.

Data on histogenesis of bone tissue and regeneration, calcium metabolism as well as on hormonal regulation of bone growth and remodeling processes are presented in this work. Recent information on the effect of the main endocrine disruptor classes (diethylstilbestrol, organochlorine pesticides, alkylphenols, bisphenol A, dioxins, polychlorinated biphenyls, and phthalic acid esters) on the development and remodeling of bone tissues and calcium metabolism has been summarized. The established physiological and molecular mechanisms of their action have been also considered.

## Introduction

The development and regeneration of bone tissues are regulated by a complex of hormones, growth factors, prostaglandins having a direct or indirect influence on the mineral exchange and the processes of cell proliferation and differentiation. The researches in this field have established that bone tissue is an active object: it is involved in numerous regulatory processes and its normal functioning is the key to maintaining the exchange of not only mineral substances but carbohydrates and lipids as well [1, 2].

The second half of the XX and the beginning of the XXI centuries became fruitful periods of studying cell biology. Cells have been determined to contain receptors perceiving information from external and internal media of multicellular organisms. Owing to these receptors, the whole complex of protective, metabolic, motor, behavioral, and homeostatic reactions necessary for the adaptation of the organism to the changing environmental conditions is triggered. Each of the reactions has been shown to have an identical structural and functional organization: reception of information by the cellular receptor apparatus, preparation to the response, and the effector action as such [3].

Hormone receptors are of special interest among cellular receptors. Each target cell has its own set of receptors which are located on its superficial apparatus (membrane), in the cytoplasm (cytosolic), or on the nuclear membrane and other cell organelles. All receptor classes possess the phenomenon of sensitivity self-regulation via a feedback mechanism. Having performed its task, the hormone is broken down in the target cell or in the blood, or is transported to the liver, where it is inactivated, or is eliminated from the organism mainly in the urine. Each hormone is known to interact with its receptors in a physico-chemical way. Conformational changes of the receptor protein take place and a complex of the signaling molecule is formed activating the receptor. Only in this state, it can cause specific intracellular reactions in response to the received signal. However, it has been found that some hormone receptors are capable of binding to other chemical compounds having structural similarity with natural ligands and cause the response of the endocrine target cell by releasing a specific hormone or blocking the signal. Thereby not only the functional activity of these cells is impaired but a hormone regulation of the whole organism as well. These substances were called disruptors, i.e. endocrine destructors.

Unfortunately, there is no unified pathogenetic definition of endocrine disruptors so far. According to one of them [4], endocrine disruptor is an exogenous agent which prevents synthesis, secretion, metabolism, transport of a specific product of the target cell that eventually impairs the regulatory mechanisms of living organisms responsible for maintaining their homeostasis. The World Health Organization [5] defines endocrine disruptor as an exogenous substance or a mixture of various substances which alters functions of the endocrine system and consequently causes adverse health effects on a human organism or its progeny. The American Association of Clinical Endocrinology [6] considers endocrine disruptors as exogenous (non-natural) chemical compounds or their mixture which affect any aspects of hormone action.

Endocrine disruptors are widely used in agriculture and industry contributing much to environmental pollution. It was found that the period of their decay may last for many decades and the products of their chemical degradation can also bind to the receptors of hormones to which the target cells respond specifically. Thus, mankind has faced a new challenge and a novel scientific direction appeared in the modern endocrinology — the study of biological impact of low-dose endocrine disruptors, previously considered biologically safe, on the processes of prenatal and postnatal development of living organisms [7].

Diverse classes of chemical substances are referred to the known endocrine disruptors: aromatic organochlorine compounds, alkylphenols, bisphenol A, diethylstilbestrol (DES), dioxin, dioxin-like components, phthalate esters (PE), benzopyrenes, polychlorinated biphenyls, and others [7, 8]. It is important to underline that they are present in varying amounts in water, air, and foodstuffs. However, their disruptor effects are not studied well enough. The investigations conducted have established that low doses of endocrine disruptors affect negatively not only endocrine system functioning but its development and immune defense as well, interfering with epigenetic regulation of morphogenetic processes [9–12]. In addition to these studies there appeared data on the effect of endocrine disruptors on skeleton tissues that results in the disorders in the locomotor apparatus which are accompanied by serious complications (osteopenia, osteoporosis, bone fractures, prolonged disability, and a high mortality rate [13].

Statistical data regarding the incidence of bone and cartilaginous tissue diseases demonstrate their growth not only in the developed countries but in the whole world [14–16]. The morbidity rate among the population of the Russian Federation shows that the diseases of the locomotor system occupy steadily the third place in its structure after cardiovascular and oncological pathologies [17]. The existing information does not allow underestimation of endocrine disruptor contribution to the development of musculoskeletal pathologies. But at the same time, a poorly studied aspect of this problem hinders the possibility of elaborating a scientifically grounded methodology for effective treatment and prevention of these diseases and demands further investigations aimed at the creation of a conceptual base for solving this important medical and social problem.

## Osteogenesis and its regulation

The development and functioning of skeletal bone tissues are known to be a very complicated hormone-dependent process regulated by numerous endocrine glands using different hormones of the thyroid gland (triiodothyronine, thyroxine, calcitonin); parathyroid glands (parathormone); adenohypophysis (somatotropin and somatostatin); adrenal cortex; reproductive glands (testosterone, estrogen, progesterone, and others). Each hormone has its own receptors in bone tissue cells and regulates definite processes of their histogenesis and further functioning of the mammalian and human skeletal bones.

Two types of bone tissues are distinguished: woven and lamellar (fine-fibered). They consist of two main cell differons: the first are osteoblasts and their aging forms (osteocytes); the second are osteoclasts. Both cell differons differ from each other by a structure, a set of receptors, and functions performed. Osteoblasts originate from the blood stem cells and are producers of bone extracellular matrix components. As osteoblasts age, they lose these functions and change into the low-active osteocytes. Osteoclasts are the derivatives of a macrophage cell line. They are characterized by a simplastic structure: they contain numerous nuclei and lysosomes in the cytoplasm which carry proteolytic enzymes destroying the elements of bone extracellular matrix, i.e. play the role of osteoblast antagonists. Only coordinated interactions of these differons provide the processes of bone tissue remodeling during their dynamic reorganization.

The process of bone tissue development, osteohistogenesis, is subdivided into two types: direct (bone development from mesenchyme) and indirect (bone development at the site of the cartilage). Direct osteohistogenesis provides formation of a woven bone tissue, while indirect one forms a cartilaginous model of the future bone with subsequent destruction and generation of endochondral bone on its remnants, and also with further reorganization into a lamellar bone tissue. Direct osteohistogenesis, which provides the opportunity to define more clearly its stages and the possible role of hormones at all stages of bone formation, is most demonstrative for understanding the mechanism of bone morphogenesis.

In the process of direct osteohistogenesis, three stages are conventionally distinguished. The first stage is formation of skeletal islands in the mesenchyme. Mesenchymal cells lose their connection with each other, become round, and intensively divide by mitosis. As the result, a dense cell aggregation is generated in the mesenchyme — a skeletogenous island. Blood vessels grow into it (the stage of proliferation and vascularization).

The second stage is the differentiation of the cells of the skeletogenous island into osteoblasts. They form a preosseous matrix (osteoid) consisting of the collagenous fibers and osseomucoid (amorphous matter).

The third stage involves mineralization of the bone matrix by calcium salts, phosphorus, and microelements. The basic mineral elements of the extracellular matrix are calcium, phosphorus, and a complex of microelements, but calcium plays the most important role in its density. It is one of the most common chemical elements of the human organism which participates in synaptic transmission of the nerve pulse, cell differentiation and death, muscular tissue contraction, blood coagulation, realization of the immune response, and other vital functions of living organisms [18]. Calcium concentration in the blood is tightly controlled and cannot change by more than 3%. A total level of blood calcium is normally in the range from 2.1 to 2.6 mmol/L being the sum of: a) complexed calcium bound to bicarbonates, lactate, citrates, phosphates (7%); b) complexed calcium bound to plasma proteins (albumins) (46%); c) ionized calcium fraction (about 47%) [19]. In recent years, the notion of regulation of calcium and phosphor exchange and the processes of bone formation and remodeling has widened. Along with the well-known regulators (parathormone, calcitriol, and calcitonin), involvement of other hormones classes in the above processes has been established, expanding in its turn the number of targets for endocrine disruptor action.

Nowadays, there is no concept of regulating each of the bone development stages. There are numerous scattered data on the role of different hormones in direct or indirect osteogenesis and bone regeneration. Much of this information is based on the analysis of changes observed in patients suffering from hypo-or hyperfunction of the endocrine glands. These investigations show that the reduction of the thyroid hormone level below the physiological level impairs bone tissue remodeling, increases the level of blood calcium while the restoration of euthyroidism accelerates bone remodeling [20]. Glucocorticoids inhibit proliferation and differentiation of osteoblasts and enhance osteoclast activity, decrease osteoprotegerin synthesis, reduce calcium absorption in the intestine, stimulate its elimination in urine, and also inhibit the secretion of sex steroids causing thereby bone resorption. Progesterone, another steroid hormone, is a glucocorticoid antagonist. It also exerts a direct stimulating effect on the formation of bone tissue via its own receptors in osteoblasts and osteoclasts [21]. Estradiol prevents the resorption of bone tissue, enhances osteoprotegerin expression by osteoblasts, and increases calcium absorption in the intestine. Testosterone plays the same role in males [21]. Somatotropic hormone also has a proestrogenic effect [22].

## Calcium exchange and its role in bone formation

Since endocrine disruptors are capable of disturbing calcium metabolism, it is important to know the ways of its intake and elimination from the body. The bulk of calcium is stored in bone tissues and serves as a buffer for ions circulating in the bloodstream. Calcium exchange constantly occurs between the bone matrix and extracellular fluid. A daily exchange can exceed 500 mmol of the mineral [18].

Three states of intracellular calcium are distinguished: contained inside the cell organelles; bound to anion or the molecule of cytoplasmic protein; free (ionized). The daily need of calcium for an adult is 20.0–37.5 mmol. The bulk of it is supplied with food by transcellular and paracellular absorption in the small and large intestine. During transcellular absorption, calcium passes through the epithelial calcium channels of the apical membrane of enterocyte microvilli.

Calbindins (proteins with high affinity to calcium) help calcium to move from the apical part of the plasmalemma to the basal-lateral membrane, and calcium transport into the blood is realized across the basal-lateral membrane with the help of ATPase and Na^+^/Ca^2+^ exchanger [23]. Paracellular calcium transport occurs through tight contacts of the mucous membrane epitheliocytes, along the entire intestine, and depends on the pH level in each of its segments. The absorbed calcium passes to the general circulation and further to various tissues of the body. The bulk of calcium is accumulated in bone tissues increasing bone mineralization. Calcium and phosphor form hydroxyapatite crystals providing supporting and trophic function of the skeleton. Another important component of the bone extracellular matrix is a soluble calcium phosphate which plays the role of a labile reserve of calcium and phosphor ions for realization of all processes of the body internal exchange. Calcium is excreted from the organism by the large intestine and kidneys and is also a hormone-dependent process [23].

## Possible mechanisms of endocrine disruptor effect on bone tissue

Endocrine disruptors cause various alterations in the metabolism of bone tissues and in their structural and functional organization. A special danger comes from the disruptors impairing the realization of estrogen effects since it is just estrogens that play one of the key roles in bone development and mineralization. The regulating function is performed mainly due to the binding to the receptors of α and β estrogens expressed both by osteoblasts and osteoclasts. The potential mechanisms of estrogen effect on bone tissue are being actively studied since their detection is of great importance for understanding the etiology and pathogenesis of the diseases of the human osteoarticular apparatus.

There is a supposition [24] that there exists a feedback in the skeleton–sex hormone system, i.e. not only hormones influence bone skeleton formation but bones impact the synthesis of the sex hormones. The authors have found that osteoblasts increase the activity of the Leydig interstitial cells secreting a male hormone testosterone. And here a question arises whether the cellular elements of the bone tissue are self-producers of the hormones and what functions are realized by these hormones. In recent years, bone tissues began to be considered to possess an endocrine function. Osteocalcin, produced by osteoblasts is an active candidate for the role of their own hormone. It is the osteocalcin that represents a biologically active substance which regulates testosterone synthesis as well as insulin and adipokine secretion [25, 26]. When some works confirming the role of the bone tissue in providing fertility were published, there appeared a figure of speech such as “males reproduce themselves by the bones”.

However, it remained unknown whether cellular elements of bone tissues were direct targets of endocrine disruptors. It was also important to make clear which endocrine disruptors interfere with realization of estrogen effects. For the last 15 years, a great deal of screening, monitoring, and experimental studies have been performed devoted to the impact of endocrine disruptors on bone tissue and their role in the development of musculoskeletal disorders which made it possible to define manifestations of disruptor action for separate classes of chemical compounds.

***Diethylstilbestrol (DES)*** is a synthetic analog of estrogens whose biologic activity is several times higher than that of estradiol. DES was used as a medication for ovarian insufficiency and after ovary removal surgery, to treat cancer of prostate, mammary glands, to correct the endocrine profile in menopause, and to prevent miscarriage. In agriculture, it was used as a supplement facilitating animal growth. However, such intensive application of DES has led to the awareness of the fact that this preparation has in many cases severe consequences (development of malignant and benign tumors, mastopathy, endometritis [27]. It has been found to cross the placental barrier. Later young women born to mothers receiving DES during pregnancy had clear cell carcinoma of the vagina which was revealed even in the third-generation progeny [28]. Boys developed significantly more often some abnormality of the reproductive system (cryptorchism, hypospadias) [29]. Since 1970 the application of DES was banned in many countries. This preparation became a kind of prototype for investigators giving them the opportunity to study the mechanisms of action of endocrine disruptors with proestrogenic effect on bone tissue, and a model for investigating the effects of estrogens themselves as it possesses their properties, their influence on structural and functional bone homeostasis without additional side effects.

Investigations carried out in the 90s showed that girls whose mothers received DES during pregnancy were noted to have increased bone mass and at the same time shortening of the tubular bones [30] which designates deceleration of growth and acceleration of ossification in the postnatal development. The experimental data [31, 32] have confirmed that DES affects bone metabolism and skeleton formation. Experiments carried out on female and male mice showed that four-week DES introduction at the dose of 500 μg/kg enhances formation of the trabecular bone in the medullary canal of the proximal femoral metaphysis and breastbone in all males but not in females. The mice developed bone lesions due to the change of the osteoblast activity that resulted in fibroplasia. Fibroplasia and overgrowth of the bone trabecular structures in the medullary canal became the loci of osteosarcoma development in case of further ongoing exposure to DES which demonstrated not only the carcinogenic effect of this substance but pointed at the factors increasing the risk of bone tumor development. The analysis of the literature data has established that DES exerts a disrupting action on the development of the tubular bones of the limbs and vertebra but this effect has a marked gender difference consisting in the elongation of the tubular bones and concurrent reduction of bone tissue density in females and bone shortening in males.

### Dichlorodiphenyltrichloroethane (DDT)

Organochlorine pesticide DDT and its metabolites are the most common endocrine disruptors on the planet [5]. Its wide use in the last century and renewed application as the main substance against the carriers of malaria, leishmaniasis, and trypanosomiasis caused its persistence in all ecosystems of the planet, especially in the world ocean [33–35]. The majority of people worldwide have been exposed to a low-dose DDT and its analogs until now since it is detected in the foodstuffs of vegetable and animal origin. DDT is accumulated in adipose tissue, therefore meat, poultry, eggs, cheese, butter, and milk may be referred to the food products containing the highest DDT levels. DDT remains a widely spread pollutant and its levels may be significant in those places where production and application of DDT are still going on or where it has been previously produced [36]. The investigations performed in 2013–2016 in the EU countries have found the presence of DDT metabolites in 100% of the examined children, and DDT itself in 80% of cases, designating the ongoing supply of this disruptor into the organism [37]. The elevated exposure to DDT and its metabolites is observed in the north countries as these compounds can be carried by the air stream over large distances, i.e. they get to the atmosphere in warmer regions and fall out to the earth surface in colder ones. In reality, the level of DDT in the Eskimo’s organisms is nearly the same as that of the people living in the regions where DDT is used to struggle against malaria [38].

The screening investigations show that there is a relation between the DDT content in the organism and bone density [39]. It has been found that a daily intake of fish and seafood which accumulate DDT to the highest degree due to a high content of lipids and a more intensive pollution of water resources, causes the development of marked osteoporosis and consequently bone fractures in women living in Northern Europe [40].

The mechanisms of disruptor action of DDT and its metabolites on bone tissues are not established but taking into consideration their ability to impair the production of the thyroid hormones [41], mineralocorticoids [42], as well as their antiandrogen and proestrogen effects [42, 43], it is possible to suggest that DDT affects indirectly the mineral exchange, including calcium exchange as well, disturbing the signaling of the mentioned hormones. These suppositions are confirmed by the results of population studies which revealed an inverse relation between the content of DDT and its metabolites and vitamin D concentration in the blood serum of the USA population [44, 45]. However, these correlations are not specific for DDT. They are also detected when other endocrine disruptors are tested in the blood or urine [46, 47]. These data require thorough experimental investigations as they indicate the possible mechanism of endocrine disruptor action. The deficit of vitamin D in the residents of Asian countries, where the insolation level is rather high, but where organochlorine pesticides are still widely used, gives evidence in favor of these assumptions [48, 49]. DDT penetrates the placental barrier and exerts a dysmorphogenetic action due to the disorder of transcriptional regulation of cell proliferation and differentiation [11, 12]. DDT is likely to affect not only the endocrine glands but other organs and tissues as well.

***Alkylphenols*** are synthetic nonionic surfactants used in the production of plastics, dyes, detergents, and pesticides. They are in abundance in the environment, found most often in water and bottom deposits of the water basins. These substances are readily absorbed in the gastrointestinal tract, possess estrogenic activity, and are endocrine disruptors inhibiting spermatogenesis and testosterone secretion [43, 50]. The experiments *in vitro* [51] have established that alkylphenols inhibit osteoclast development but do not affect cell proliferation, differentiation, and mineralization of the osteoblastic population. Their impact (0.1 μg/kg) on the pregnant mice at days 10 to 14 after mating accelerates ossification of the fetus sternum segments. This is considered to be a consequence of osteoblast formation inhibition leading to skeletal malformation. Organism development exposed to lower doses of alkylphenols in prenatal and early postnatal periods results in osteocalcin synthesis and malformation of bone diaphysis without changes of the bone length in female mice. The authors believe [52] that decrease of alkaline phosphatase synthesis and periosteal osteoblast number is likely to be the mechanism of such development. It has been shown in the experiments *in vitro* [53] that introduction of micromolar solutions of nonylphenol to the culture of alvarial osteoblasts causes their apoptosis which develops via the external and internal (mitochondrial) pathway and this distinguishes alkylphenols from organochlorine pesticides.

***Bisphenols*** are a group of synthetic monomers widely used in industry as a plastic curing agent. Bisphenol A, the basis of epoxy resin and polycarbonate plastic, is a representative of this class which is most frequently used. It is applied in the production of containers for food products, disposable cutlery, toys, sports equipment, thermal paper for cash register tapes, CDs and DVDs, household appliances, etc. This substance is also used for manufacturing medical instruments and goods, and especially widely in dentistry as it serves as a basis for the greater part of modern composite materials [54–56]. Food products became the most common source of bisphenol A for people [57]. In this connection, in some countries of the European Union, bisphenol A was first banned for the production of children tableware and packages for baby food and then in the production of packages for other food products [58].

Biologically, bisphenols are xenoestrogens. They are capable of binding to the nuclear and membrane estrogen receptors and activate rapid cascades of target cells [59]. Besides, bisphenol compounds have demonstrated the ability to interact with androgen and glucocorticoid receptors [60] and disturb thyroid hormone signaling [61]. The danger of bisphenol A is primarily connected with its ability to pass across the hematoplacental barrier and affect intrauterine development of the rat embryos and fetuses including their metabolism and formation of skeletal bones [62]. High doses of this substance (over 100 mg/kg) induced toxic effects and ossification disorder.

However, it is necessary to differentiate distinctly toxic and disruptor action of the substance. The results obtained in the studies like this cannot be considered to prove the properties of endocrine disruptor impairing bone tissue metabolism. Endocrine disruptor impairs hormonal regulation affecting the organism in the amount similar to endogenous hormones. Experiments on the effect of bisphenol A on the pregnant rats in the doses equal to the level of the natural exposure of this substance to the human organism have shown that the effect started from the first day of pregnancy becomes evident mainly in the male progeny in the form of femoral bone shortening and decreased trabecular area [62]. All the above mentioned give reason to believe that bisphenol A not only affects estrogenic activity but interferes with the processes of bone skeleton formation in the prenatal period.

Interesting data were obtained during examination of women with diagnosed osteoporosis in the postmenopausal period [63]. A direct relation between the bisphenol level and blood calcium concentration was detected which was likely caused by the proestrogenic effect. Other investigations [64] have shown the reduction of calcium concentration in the blood due to inhibition of calcitonin secretion. However, data obtained during examination of human individuals are not reliable enough as mankind is constantly subject to the impact of hundreds of chemical and physical factors and the detected correlations are referred to the category of probable and tentative for data investigators.

***Dioxins*** are one of the most stable pollutants on the planet. Their life-half period ranges from 7 to 11 years. They enter the organism with air, food, and water. Dioxins possess a strong immunosuppressive, mutagenic, carcinogenic, and embryotoxic properties [65–67]. Dioxins and dioxin-like pollutants are highly lipid-soluble, they accumulate in the human and animal organism in lipocytes of the adipose tissue. They are poorly metabolized and slowly excreted from the organism. In everyday life, dioxins are produced when burning plastic tableware and other garbage with a high content of organochlorine compounds. The main danger of the biological effect of dioxin and its derivatives consists in their ability to bind to the cellular receptors and inhibit or alter their physiological properties. The effect of dioxin and dioxin-like compounds on bone tissues is not sufficiently studied and data from the published sources do not provide the opportunity to make a scientifically grounded conclusion on the general principles of their influence on metabolism, signal pathways, structural and functional organization of bone tissues.

Among dioxins, 2,3,7,8-tetrachlorodibenzo-p-dioxin (TCDD) possesses the strongest biological action. It is able to bind to aryl hydrocarbon receptor (АhR), one of the most ancient phylogenetic highly conservative transcription factors existing for more than 600 million years. Binding of TCDD to АhR results in its translocation into the nucleus and transcription of dioxin-dependent genes [68]. One of the AhR functions is to increase the activity of enzymes breaking down xenogenic substances and hydroxylate estradiol [69]. AhR is also expressed by osteoblasts and osteoclasts most actively after the stage of matrix hardening and before the beginning of mineralization in the differentiating osteoblasts [70].

There are data that AhR is involved in osteogenesis and regulates the synthesis and metabolism of estrogens in bone tissues [71]. The *in vitro* experiments [72] have shown formation disorder of multicellular nodular structures by calvarial osteoblasts exposed to TCDD, the reduction of collagen type I synthesis, alkaline phosphatase activity, and mineralization of stromal medullary cells. These results give evidence that AhR ligands inhibit differentiation in the osteogenic direction and osteogenesis. The experiments *in vivo* on the rat lines with different sensitivity to TCDD have established both bone malformation (a shorter bone length) and a dose-dependent effect of this change [73]. Using a mice and rat model of differentiation of tibia stromal medullary cells it was confirmed that the TCDD disrupting action on bone development and regeneration consists in the decrease of the alkaline phosphatase activity, expression of osteocalcin and bone morphogenetic protein 2, and inhibition of osteoclast differentiation [74].

The study of dioxin effect at different stages of ontogenesis has shown that there are periods most vulnerable to TCDD impact [75]. The intrauterine period does not affect essentially the development of the limb bones while they exhibit high sensitivity to this endocrine disruptor in the breastfeeding period. The effect of dioxins on the developing organism leads to the disorder of direct and indirect osteogenesis. Rats exposed to TCDD during their development have been noted to have reduced sizes of the skull with predominant malformation of the facial bones in females. The bones of the facial and brain part of the skull manifested the greatest sensitivity to TCDD in the prenatal period and during milk-feeding but not in the later developmental period [75].

***Polychlorinated biphenyls (PCBs)*** are a class of organic compounds distinguished by their high chemical stability. The main fields of application are electrical engineering where they are used as insulators, retardants, lubricants, hydraulic fluid, and in pesticide production. Similar to DDT these compounds possess high lipophilicity, ability to accumulate in the food chain, and slow biodegradation [76]. Although their use is prohibited, TCDDs continue to be one of the widely distributed stable organic pollutants [6, 77]. Their disrupting action is established to affect thyroid function. They impair expression of thyrotropin-dependent genes, are capable of binding to transthyretin disturbing not only secretion of the thyroid hormones but their transport in the general circulation as well [78]. One of the main mechanisms of the TCDDs disrupting impact, like in the case with PCBs, is the ability to bind to AhR [79]. Exposure to PCBs causes different cognitive disorders of the developing organism during the antenatal period, disorders of bone tissue development were also noted [80]. Monitoring of the postnatal development of children whose mothers consumed food products containing PCBs has revealed shortening of the skeletal bones, abnormal calcification of the skull bones, and retarded dentition [5].

***Phthalate esters (PE)*** are esters of phthalic (orthophthalic) acid. About 10 million tons of PEs are produced annually worldwide. They are components of polyvinyl chloride production and are used as plasticizers in various branches of industry including healthcare and food industry [81]. PEs possess a low degradation level but are considered as nonpersistent pollutants. Their effect on the human organism is mainly connected with their content in food products and cosmetic goods. A wide application of PEs in industry resulted in significant contamination of soil and inland water bodies in the countries of Europe and Asia as well as seas, and this allows the revision of the existing notion about the levels of PEs impact [82–85]. These substances are referred to the compounds with estrogen-like effect due to binding to estrogen receptors, they also are capable of competing with androgens for binding to a receptor [86]. They impair not only functional activity of the reproductive systems in humans and animals but other glands of internal secretion as well causing hormonal disbalance in regulation of living organism functions [87–89]. For this reason, PEs are considered to be endocrine disruptors. Embryotoxic PEs effect was also detected [90–92]. Besides they were determined to bind to AhR [93].

At the same time, the disrupting PEs effects on bone tissues are not studied fully enough. The experiments *in vitro* conducted on the rat osteoblasts [94] have shown that PEs effect in nanomolar concentrations modifies intracellular localization of fibroblast growth factor 2 which is one of the regulators of bone remodeling. Exposure of the developing organism to PEs causes abnormal skeleton development. In contrast to dioxins, PEs affect negatively vertebra, ribs, sternum, and maxillofacial bones. Animals exposed to DDT in the prenatal period were often noted to have abnormal fusion of the vertebral arches, sternum segments, and cleft palate formation [95]. PEs impact in the prenatal and early postnatal periods was found to impair later bone tissue remodeling [96]. The investigations show that one of the mechanisms of PEs disrupting action is osteoblast differentiation disorder [97]. Screening data of the recent years demonstrate the link between low-dose PEs effect and the development of osteoporosis in women in the postmenopausal period [98].

The experimental studies have shown that the main mechanism of osteoporosis under the condition of estrogenic deprivation is the PEs-induced reduction of osteocalcin production [99]. Impaired synthesis of vitamin D is considered as a second pathogenetic link in the development of osteoporosis caused by PEs exposure, however, information on this problem is controversial [47, 100, 101].

## Conclusion

Data available to date regarding endocrine disruptors provide evidence that they interfere with formation and regeneration of bone tissues by destabilizing the balance of proliferation, differentiation, and functioning of osteoblasts and osteoclasts and also alter the parameters of calcium metabolism due to the impairment of secretory activity of the endocrine glands and hormone signaling (see the Figure). It should be noted that children and adolescents are highly sensitive to their effect, i.e. these periods of ontogenesis should be considered as critical periods of organism development and all available measures should be taken in order to limit the influence of endocrine disruptors both *in utero* and postnatal development. No doubt, that endocrine disruptor impact on the developing and adult organisms causes changes which create the basis for the musculoskeletal pathology development and must be considered as risk factors, on the one hand, and as etiological factors of this group of diseases which are to be studied since a specific character of the disruptor action may minimize the efficacy of medical procedures.

**Figure:**
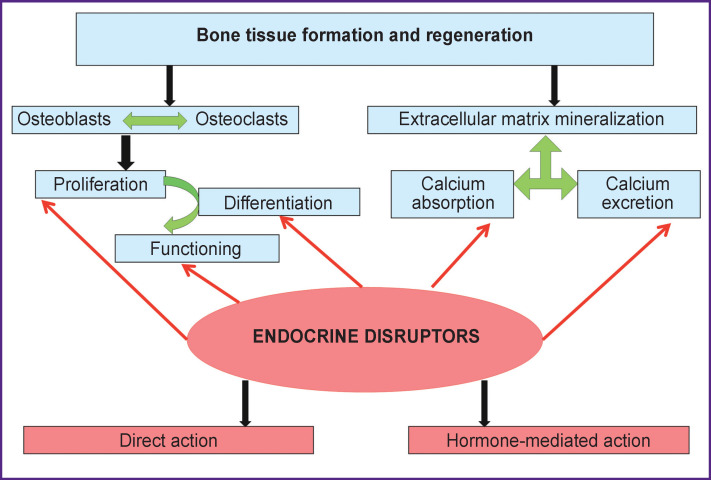
Main targets and mechanisms of dysregulation action of endocrine disruptors interfering with the development and regeneration of bone tissue (the authors’ drawing)
